# miRNAs that associate with conjunctival inflammation and ocular *Chlamydia trachomatis* infection do not predict progressive disease

**DOI:** 10.1093/femspd/ftx016

**Published:** 2017-02-08

**Authors:** Tamsyn Derrick, Athumani M. Ramadhani, Karim Mtengai, Patrick Massae, Matthew J. Burton, Martin J. Holland

**Affiliations:** 1 Clinical Research Department, London School of Hygiene and Tropical Medicine, London WC1E 7HT, UK; 2 Kilimanjaro Christian Medical Centre, PO Box 3010, Moshi, Kilimanjaro, Tanzania

**Keywords:** *Chlamydia trachomatis*, trachoma, fibrosis, miRNA, biomarker, inflammation

## Abstract

We previously showed that conjunctival miR-147b and miR-1285 were upregulated in Gambian adults with inflammatory scarring trachoma, and miR-155 and miR-184 expression was strongly associated with conjunctival inflammation and ocular *Chlamydia trachomatis* infection in children from Guinea-Bissau. We investigated whether the single or combined expression of miR-147b, miR-1285, miR-155 and miR-184 was able to identify individuals with increased risk of incident or progressive scarring trachoma. Conjunctival swab samples were collected from 506 children between the ages of 4 and 12 living in northern Tanzania. These 506 samples formed the baseline sample set of a 4-year longitudinal study. *Chlamydia trachomatis* infection was diagnosed by droplet digital PCR and expression of miR-155, miR-184, miR-1285 and miR-147b was tested by qPCR. Individuals were assessed for incidence and progression of conjunctival scarring by comparison of conjunctival photographs taken at baseline and 4 years later. miR-184 and miR-155 were strongly associated with inflammation and infection at baseline; however, no miR was associated with 4-year scarring incidence or progression. miR-184 expression was more strongly downregulated during inflammation in non-progressors relative to progressors, suggesting that a disequilibrium in the efficiency of wound healing is a significant determinant of progressive conjunctival fibrosis.

## Teaser

miRNA associated with ocular *Chlamydia trachomatis* infection and clinical inflammation do not predict progressive scarring disease.

## INTRODUCTION

Trachoma is a fibrotic disease, initiated by conjunctival infection with *Chlamydia trachomatis* (Ct) during childhood. It is currently estimated that 3.2 million people suffer with trachomatous trichiasis and are at risk of sight loss (World Health Organization 2016).

Micro RNAs (miR) are post-transcriptional regulators of gene expression. Individual miR can regulate hundreds of genes (Ambros 2004) and single or small numbers of miR can have profound roles in the regulation of biological processes in health and disease, including fibrosis (Cushing *et al.*^2011^) and inflammation (Rodriguez *et al.*^2007^; Yao *et al.*^2012^; Xiang *et al.*^2014^). In addition to aiding our understanding of the complex transcriptional patterns associated with disease mechanisms, miRNA can be used as predictors or biomarkers of disease. Previous work in the murine genital tract model of *C. muridarum* infection demonstrated that a virulent, pathology-inducing variant induced differential expression of host miR 24 h post-infection relative to a variant that induced less inflammatory pathology (Yeruva *et al.*^2014^).

It is thought that the vast majority of individuals living in trachoma-endemic communities are exposed to Ct throughout childhood and that repeated episodes of infection and associated inflammation lead to progression of scarring disease and eventually trichiasis. It is not understood why only some exposed individuals progress to scarring disease whereas others do not. A miR transcriptional signature that could classify those at risk of scarring progression before the onset of clinical signs could enhance our understanding of the disease process and could be used as biomarkers of protective or adverse effects in response to a Ct vaccine, if one was made available for testing in human populations.

We previously identified that miR-147b and miR-1285 were upregulated during inflammatory trachomatous scarring in Gambian adults (Derrick *et al.*^2013^), and that differential expression of miR-184 and miR-155 was strongly associated with ocular Ct infection and active trachoma in children from Guinea-Bissau (Derrick *et al.*^2016^). We investigated whether these four miR could predict trachomatous scarring progression.

## METHODS

This study was approved by the London School of Hygiene and Tropical Medicine ethics committee, the Tanzania National Institute for Medical Research ethics committee and the Kilimanjaro Christian Medical Centre ethics committee and it adhered to the tenets of the Declaration of Helsinki.

Conjunctival swabs were collected using standard methodology (Keenan *et al.*^2010^; Last *et al.*^2014^) from 506 children in the West Kilimanjaro region of northern Tanzania, prior to the introduction of MDA. These 506 children formed the baseline sample set of a cohort (totalling 666 children) seen every 3 months for 4 years. MDA was initiated 6 months after baseline and three annual rounds were given; the last dose was given 18 months prior to the final timepoint. High-resolution photographs were taken prior to swab sample collection. Swabs were stored in RNA*later*^®^ (Thermo Fisher Scientific, Waltham, USA) until total RNA and DNA extraction (Norgen Biotek Corp., Thorold, Canada). DNA was tested for Ct infection using a ddPCR assay described elsewhere (Roberts *et al.*^2013^). RNA was reverse transcribed using TaqMan® MicroRNA Reverse Transcription Kit (Thermo Fisher Scientific) and qPCR was performed on a VIIA7 thermal cycler using TaqMan small RNA assays (Thermo Fisher Scientific) for endogenous control U6, miR-155-5p, miR-184, miR-147b and miR-1285. To investigate the mRNA expression of matrix metalloproteinase 7 (*MMP7*) and endogenous control *HPRT1*, RNA was reverse transcribed using SuperScript® VILO cDNA synthesis kit (Thermo Fisher Scientific) and qPCR was performed on a VIIA7 thermal cycler using TaqMan Low Density Array microfluidic cards (Thermo Fisher Scientific).

Of the 506 individuals seen at baseline, 346 were seen 4 years later (31.6% loss to follow-up) and high-resolution photographs were taken. Baseline photographs were graded for clinical signs using the WHO 1981 FPC grading system (Dawson, Jones and Tarizzo 1981). For analysis of clinical signs at baseline, follicular (F) and papillary (P) inflammation scores were combined into a single inflammation score as follows: F0 and P0 = no inflammation, F1 and/or P1/P2 = mild inflammation, F2/F3 and/or P3 = active trachoma. Scarring (C) scores 2 and 3 were combined for analysis due to the small number in each group; thus, scarring groups were as follows: C0 = no scarring, C1 = mild scarring and C2/3 = moderate scarring. Baseline and 4-year photographs were compared side by side by an experienced clinical trachoma grader to identify individuals with incident (the appearance of any new scar tissue) or progressive (an increase in the amount of scar tissue) scarring (Burton *et al.*^2015^). Statistical analyses were performed in R (www.r-project.org). Thirteen samples were removed from the analysis during quality control (endogenous control U6 cycle threshold value (CT) > mean ± (2 × standard deviation)). Delta CT (ΔCT) values were calculated using U6, and 40-ΔCT values were used in the analysis to reflect the direction of expression.

## RESULTS AND DISCUSSION

At baseline, the median age was 7.5 (range 4–12) and 49.6% of children were male. 97.4% were of Maasai ethnicity; others were Chaga, Sonjo and Pare. A total of 78 individuals had Ct infection (15.4%), 152 (30%) of individuals had no inflammation, 171 (33.8%) had mild inflammation and 183 (36.2%) of individuals had active trachoma (defined by the grading system described above). No conjunctival scarring was seen in 71.9% of individuals at baseline, 23.5% had mild scarring (C score 1) and 4.5% of individuals had moderate scarring. Of the 78 Ct infections at baseline, 80.8% were found in individuals with active trachoma.

Consistent with our previous results in children from Guinea-Bissau (Derrick *et al.*^2016^), miR-1285 and miR-147b were not associated with any clinical phenotypes or infection, whereas miR-184 and miR-155 were strongly associated with inflammation and infection (Table 1). miR-155 was upregulated in inflammation and infection and miR-184 was downregulated. miR-155 has many roles in formation and development of immune cells and in regulating immune response pathways (reviewed in Vigorito *et al.*^2013^). miR-155 has recently been shown to be involved in murine bone marrow-derived dendritic cell activation following *Chlamydia muridarum* infection (Gupta *et al.*^2016^). The observed upregulation of miR-155 probably reflects the increased presence and activity of immune cells in the conjunctival epithelium and its associated lymphoid tissue during ocular Ct infection and inflammation. miR-184 is strongly expressed in the conjunctival epithelium and its downregulation is thought to activate the Wnt pathway and promote neovascularisation in response to ocular epithelial injury (Takahashi *et al.*^2015^; Zong *et al.*^2016^). miR-184 negatively regulates the Wnt receptor frizzled-7 and a miR-184 mimic inhibited Wnt signalling in a murine ocular injury model (Takahashi *et al.*^2015^). MMP7 is transcriptionally regulated by β-catenin, a Wnt signal transducer, and in malignant tissues and chronic kidney disease MMP7 expression is positively correlated with Wnt activation (Brabletz *et al.*^1999^; Crawford *et al.*^2001^; Machin *et al.*^2002^; He *et al.*^2012^). Upregulation of MMP7 and enrichment of genes in the Wnt pathway have previously been reported in individuals with scarring trachoma and trichiasis (Holland *et al.*^2010^; Burton *et al.*^2011^). We investigated whether the downregulation of miR-184 expression was correlated with an upregulation of *MMP7* expression as a surrogate marker for Wnt pathway activation. In these baseline samples however there was a marginally positive correlation between *MMP7* expression and miR-184 expression (*P* = 0.053, adjusted R^2^ = 0.006). These samples consisted of mixed cell populations, and thus correlations between miR and effector expression should be examined in single cells or epithelial cells.

**Table 1.** Multivariate linear regression results showing associations of clinical and infection status at baseline with miR expression.

	**miR-1285**		**miR-147b**		**miR-155**		**miR-184**	
	***P* value**	**OR (CI)^a^**	***P* value**	**OR (CI)^a^**	***P* value**	**OR (CI)^a^**	***P* value**	**OR (CI)^a^**
**Mild inflammation**	0.242	1.19 (0.89–1.59)	0.043	1.44 (1.01–2.05)	0.027	1.36 (1.04–1.78)	**3.44 × 10^–^^4^**	0.61 (0.47–0.80)
**Active trachoma**	0.937	1.01 (0.73–1.40)	0.156	1.33 (0.90–1.98)	**5.26 × 10^–^^5^**	**1.89 (1.39–2.57)**	**4.46 × 10^–^^8^**	0.43 (0.32–0.58)
**Mild scarring**	0.169	1.23 (0.92–1.65)	0.628	1.09 (0.76–1.56)	0.369	1.13 (0.86–1.49)	0.252	1.17 (0.89–1.53)
**Moderate scarring**	0.325	0.75 (0.43–1.32)	0.061	0.52 (0.26–1.03)	0.033	0.56 (0.33–0.95)	0.425	0.81 (0.48−1.36)
**Ct infection**	0.254	1.22 (0.86–1.74)	0.559	1.13 (0.74–1.73)	**4.22 × 10^–^^8^**	**2.53 (1.83–3.51)**	**1.72 × 10^–^^6^**	0.45 (0.33–0.62)
**Age**	**0.008**	**0.92 (0.87–0.98)**	0.583	0.98 (0.91–1.05)	0.577	0.98 (0.93–1.04)	0.028	1.06 (1.01–1.12)
**Female**	0.369	0.90 (0.71-1.13)	0.902	1.02 (0.77–1.35)	0.50	1.07 (0.87–1.34)	0.606	0.95 0.76–1.17)

a OR = odds ratio; CI = 95% confidence intervals.

Unadjusted *P* values are shown. Correcting for multiple comparisons using Bonferroni adjustment, *P* values <0.0125 would be considered significant (highlighted in bold). Data from 493 individuals (13 samples excluded from the 506 baseline samples during quality control) were included in these models. miR expression was the linear-dependent variable and clinical signs, infection status, age and sex were independent variables.

Of the 346 individuals that were seen 4 years later, 262 (75.7%) had no evidence of new scar tissue. Progressive or incident scarring was identified in 84 individuals (24.3%) and there were no individuals that showed a reduction in the amount of scar tissue.

**Figure 1.**
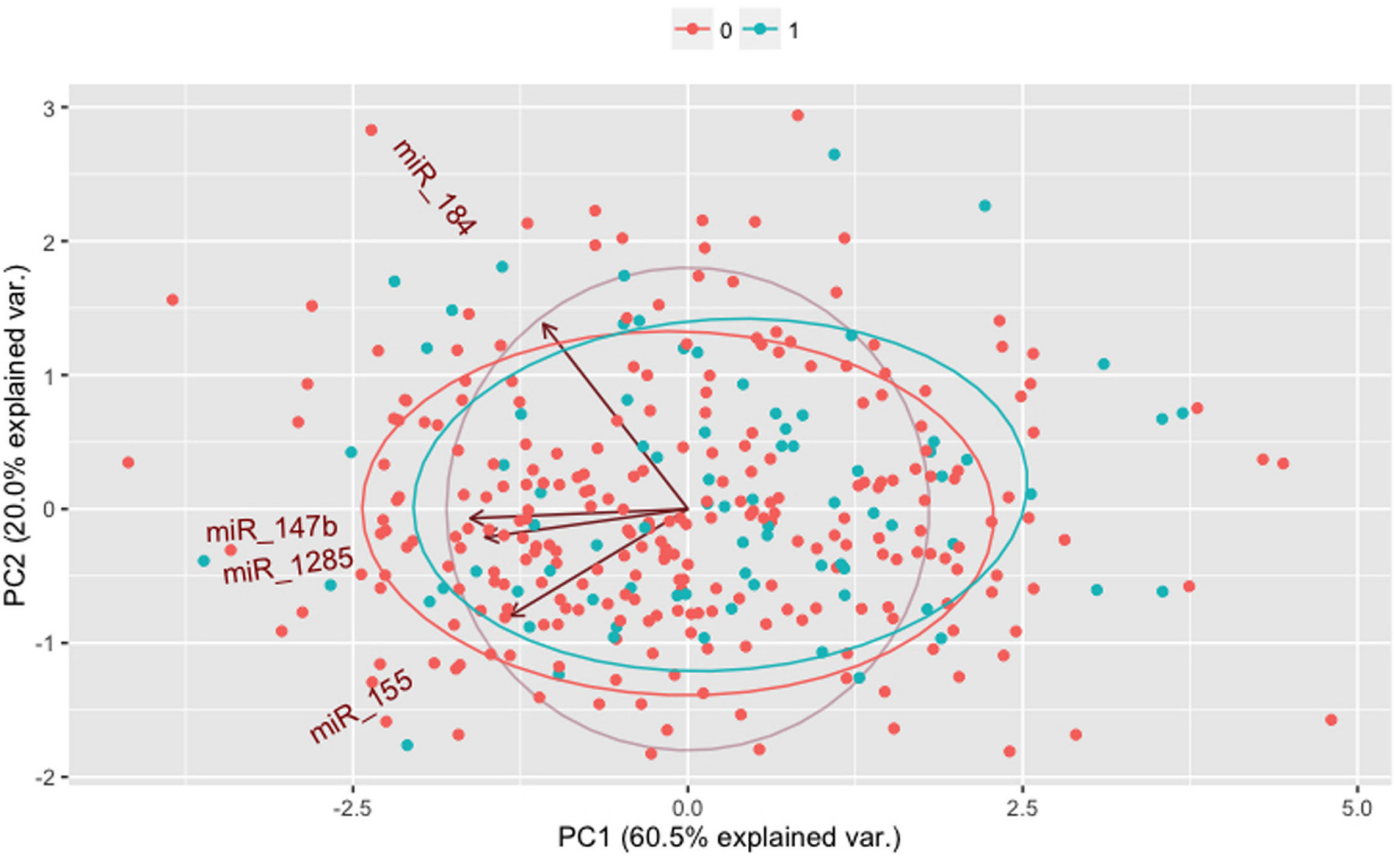
Principle component plot of the expression of miR-184, miR-155, miR-147b and miR-1285 at baseline. Scarring progressors (1; blue) and non-progressors (0; red) are overlaid.

Principle component analysis of the expression of miR-155, miR-184, miR-147b and miR-1285 did not differentiate scarring progressors and non-progressors (Fig. 1). Although there was slight separation along principle component one (PC1), it was not significant (*P* = 0.113, OR = 1.14 (0.97–1.34)). In univariate models (adjusting for age and sex), expression levels of the four miR at baseline were not associated with scarring progression (Table 2). Mild inflammation, active trachoma, and mild and moderate scarring at baseline were strongly associated with scarring progression 4 years later.

**Table 2.** miR expression and clinical signs at baseline associated with 4-year scarring progression.

	Univariate models		Stepwise best fit	
	*P*	OR (CI)^a^	*P*	OR (CI)^a^
Mild inflammation	**0.0017**	**4.21 (1.80–11.13)**	**0.0057**	**3.77 (1.54–10.34)**
Active trachoma	**2.75 × 10^−^^7^**	**10.83 (4.62–29.01)**	**1.81 × 10^−^^5^**	**8.74 (3.41–25.21)**
Mild scarring	**2.56 × 10^−^^10^**	**7.15 (3.91–13.28)**	**3.19 × 10^−^^7^**	**5.33 (2.82–10.19)**
Moderate scarring	**1.61 × 10^−^^4^**	**7.13 (2.56–20.26)**	**0.0055**	**4.62 (1.56–13.95)**
Ct infection	0.097	1.73 (0.89–3.27)		
miR-1285	0.163	1.17 (0.94–1.45)		
miR-147b	0.076	1.17 (0.98–1.41)		
miR-155	0.105	1.19 (0.96–1.47)		
miR-184	0.972	1.00 (0.82–1.23)	0.041	1.30 (1.02–1.68)

a OR = odds ratio; CI = 95% confidence intervals.

Unadjusted *P* values are shown. Correcting for multiple comparisons using Bonferroni adjustment, *P* values <0.0063 would be considered significant (highlighted in bold). Data from 338 samples were included (8 samples were excluded from the 346 individuals seen at 4 years during quality control). Scarring progression (binary) was the dependent variable. Age and sex were adjusted for in all models. The stepwise best-fit model initially included all independent variables tested in univariate analyses and removed them one by one, finally only retaining variables that best describe variation in the dependent variable.

**Figure 2.**
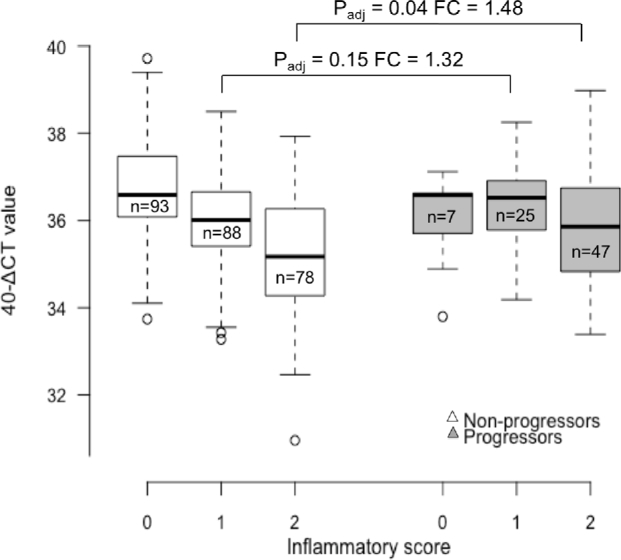
miR-184 expression was more strongly downregulated during inflammation in non-progressors relative to scarring progressors. Inflammatory score 0 = no inflammation, 1 = mild inflammation, 2 = active trachoma. Differences between groups were tested using Student's *t*-test and were adjusted using Bonferroni correction. Boxes show the interquartile range of miR expression and the bold black central line shows the median. The number of individuals in each group (n = x) is shown inside each box. Grey-filled boxes show miR expression in scarring progressors and white-filled boxes show miR expression in non-progressors. FC = fold change.

In a stepwise multivariate logistic regression model, including all miR and clinical phenotypes *a priori*, inflammation, scarring and miR-184 expression were retained as the variables that best explained variation in progression status. Interestingly, an increase in miR-184 expression was associated with increased risk of progression (*P* = 0.041, OR = 1.3). Further investigation of miR-184 expression in progressors and non-progressors, analysed by inflammatory score categories, revealed that miR-184 was more strongly downregulated in the presence of inflammation in non-progressors relative to scarring progressors (Fig. 2). Receiver-operating characteristic curves testing the ability of inflammation and scarring scores at baseline to predict scarring progression 4 years later had an area under the curve (AUC) of 0.792; however, this was not significantly improved by the addition of miR-184 expression (AUC = 0.801), or in fact by the addition of all four miR (AUC = 0.802).

These data show that the presence of scarring and inflammation at baseline, but not chlamydial infection, is associated with scarring progression 4 years later. Unfortunately, miR-184, miR-155, miR-147b and miR-1285 do not show potential to serve as biomarkers to predict individuals at risk of developing further scarring. It is possible that other miR that are not involved in the acute response to Ct infection or in late-stage scarring regulate the pathways leading to disease progression. In order to identify these miR, expression should be investigated in longitudinal samples collected along the continuum of disease. We found that scarring progressors did not downregulate miR-184 during inflammation as much as non-progressors. This might suggest that scarring progressors had weaker activation of the Wnt pathway and neovascularisation during or following inflammation, and that inefficient wound healing could contribute to chronic fibrosis. At baseline, there was no inverse correlation between miR-184 and the expression of *MMP7*, a surrogate marker of Wnt pathway activation. The expression of *MMP7* and other Wnt pathway members should also be investigated in longitudinal samples to assess the potential contribution of the Wnt pathway to disease progression. Whilst a miR biomarker for progressive scarring trachoma has so far not been identified in trachoma, further investigation of miR-184, the WNT pathway and efficiency of wound healing might inform our knowledge of the pathology of scarring trachoma.
